# Supplementary material for: Tau assemblies do not behave like independently acting prion-like particles in mouse neural tissue
Source: Acta Neuropathol Commun. 2021 Mar 12;9:41. doi: 10.1186/s40478-021-01141-6 (PMC7953780; doi:10.1186/s40478-021-01141-6)
Supplement: Supplementary file 1 — Additional file 1: Supplementary Figures. [file 40478_2021_1141_MOESM1_ESM.docx]

**
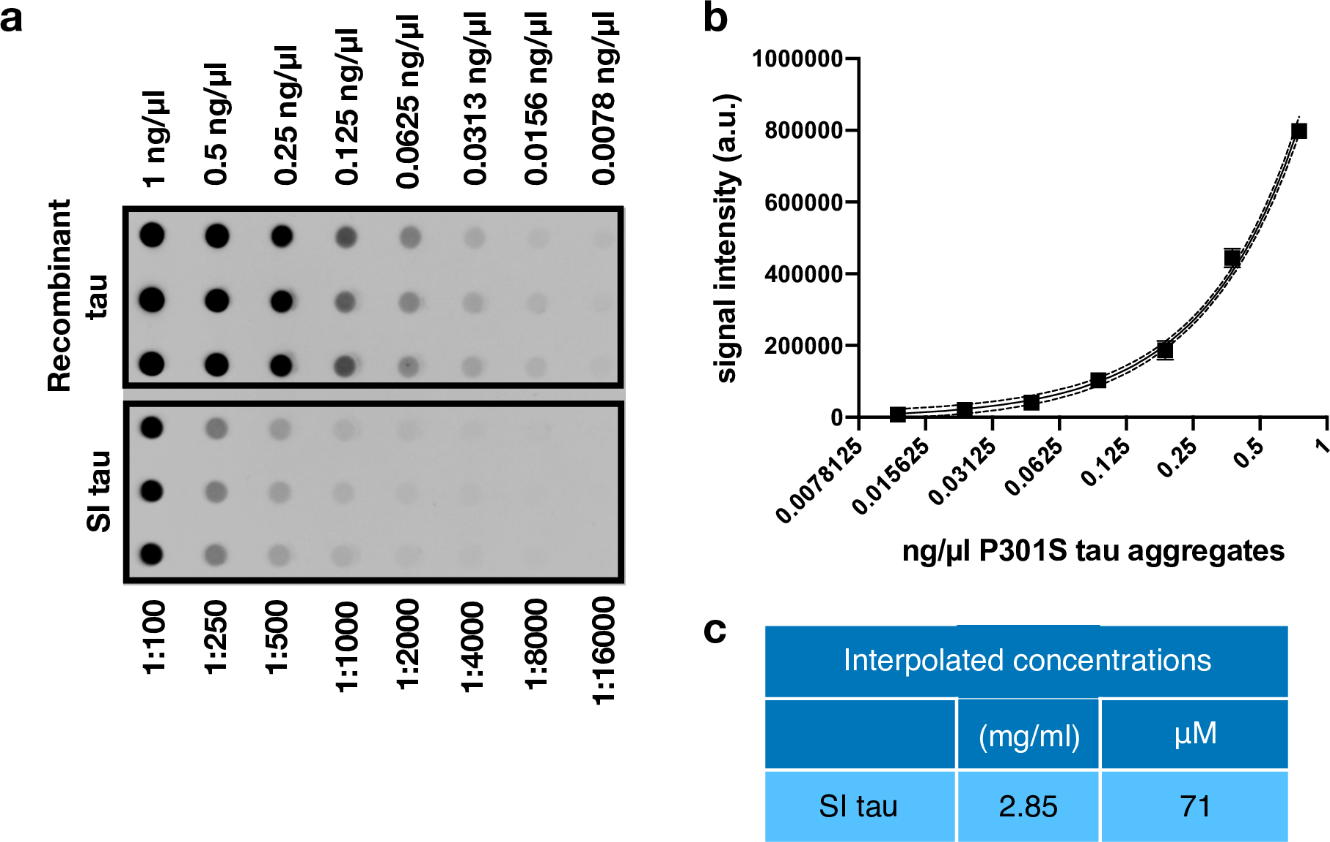
**

**Supplementary Figure 1: Quantification of SI mouse brain derived tau.**

**a.** A dot blot titration of SI mouse brain derived tau, with recombinant tau assemblies at the indicated concentrations used as a standard. **b**. Standard curve generated from dot blot signal intensities. **c.** Interpolated concentration of SI mouse brain derived tau.


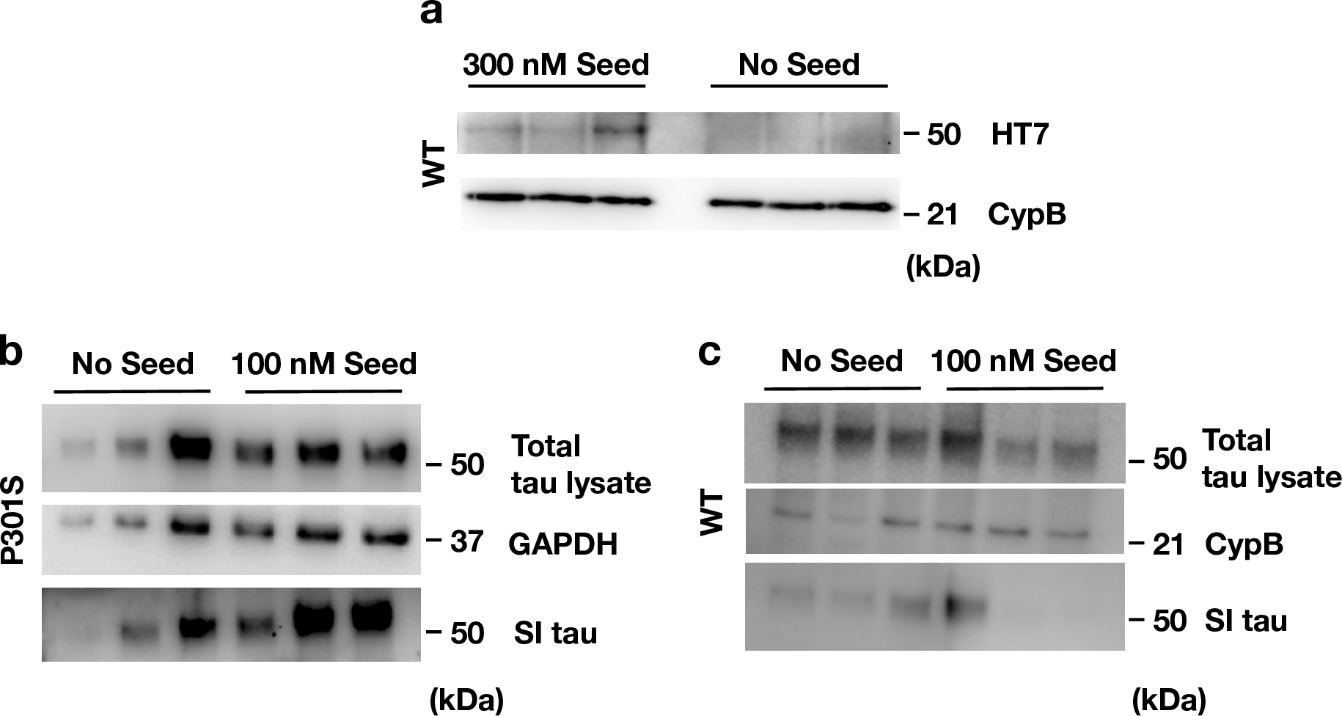


**Supplementary Figure 2: Tau uptake and SI extraction of P301S and WT OHSCs.**

**a.** Western blot of WT slices exposed to human recombinant P301S tau assemblies, probed with human-specific tau antibody HT7. **b**. Western blot of the total tau in P301S OHSC lysate and the SI fraction of P301S OHSCs with and without the addition of 100 nM recombinant tau assemblies. **c.** Western blot of the total tau in WT OHSC lysate and the SI fraction of WT OHSCs with and without the addition of 100 nM recombinant tau assemblies.


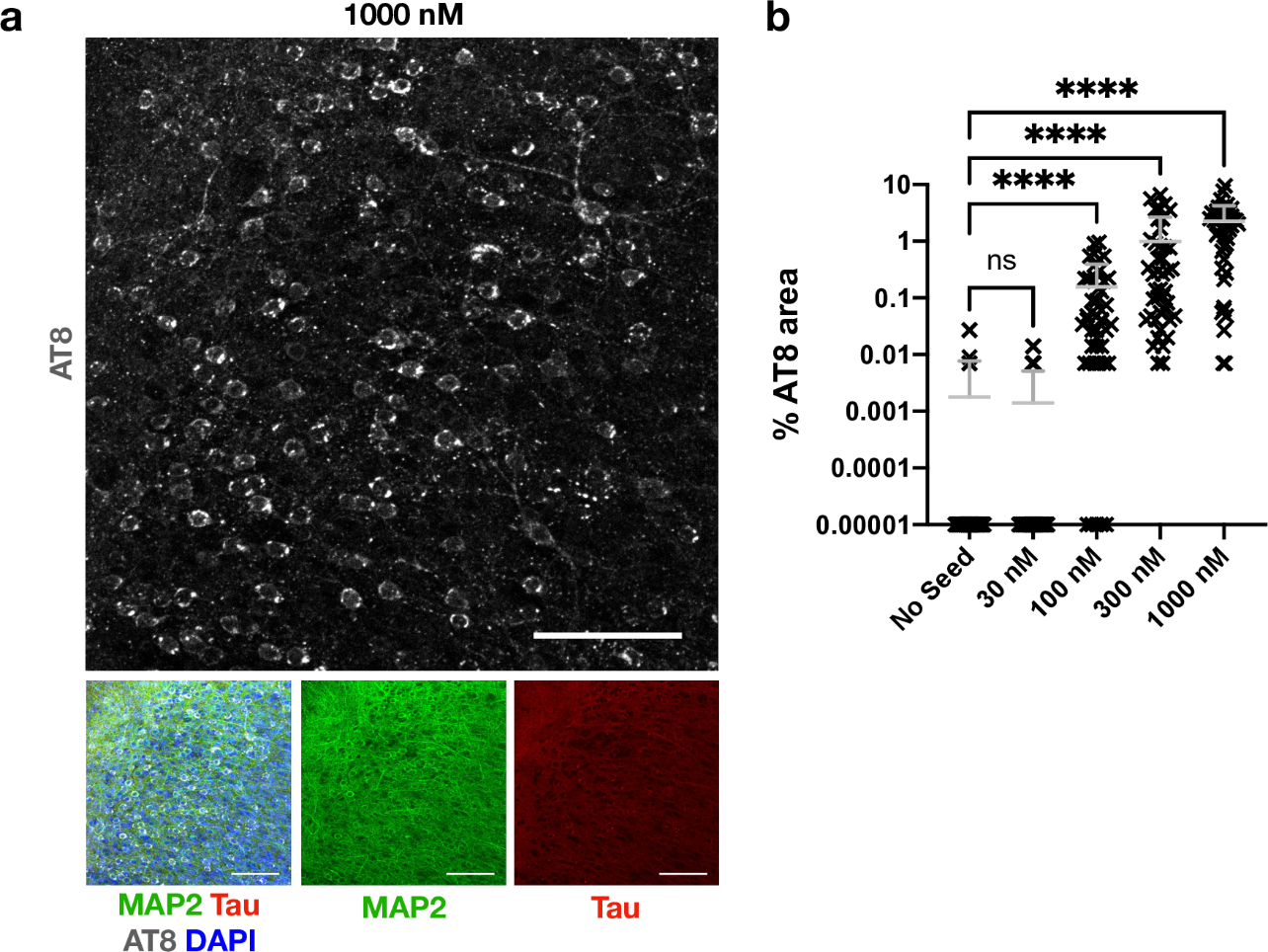


**Supplementary Figure 3: Tau seeding approaches saturation in OHSCs in the micromolar range.**

**a.** Immunofluorescent images of P301S tau transgenic OHSC three weeks after 1000 nM of recombinant P301S tau assemblies supplied to the apical side. **b**. Quantification of seeding levels in P301S OHSCs, upon the addition of recombinant tau assemblies or buffer only to the apical surface of individual slices. Statistical significance determined by Kruskal-Wallis Test by ranks and Dunn’s multiple comparisons test (Slices from N=3 mice per condition. **** P<0.0001).

**Supplementary Table 1: Summary of reported concentrations of tau used in stereotaxic injection experiments showing seeded aggregation of tau.**

Stereotaxic injection experiments separated by the source of tau used. Molar concentrations calculated from the types of tau used. The average molecular weight of the six tau isoforms was used in the case of human brain extract.

**Supplementary References**

1. Iba, M. *et al.* Synthetic Tau Fibrils Mediate Transmission of Neurofibrillary Tangles in a Transgenic Mouse Model of Alzheimer’s-Like Tauopathy. *J. Neurosci.* **33**, 1024 (2013).

2. Peeraer, E. *et al.* Intracerebral injection of preformed synthetic tau fibrils initiates widespread tauopathy and neuronal loss in the brains of tau transgenic mice. *Neurobiol. Dis.* **73**, 83–95 (2015).

3. Boluda, S. *et al.* Differential induction and spread of tau pathology in young PS19 tau transgenic mice following intracerebral injections of pathological tau from Alzheimer’s disease or corticobasal degeneration brains. *Acta Neuropathol. (Berl.)* **129**, 221–237 (2015).

4. Guo, J. L. *et al.* Unique pathological tau conformers from Alzheimer’s brains transmit tau pathology in nontransgenic mice. *J. Exp. Med.* **213**, 2635–2654 (2016).

5. Iba, M. *et al.* Tau pathology spread in PS19 tau transgenic mice following locus coeruleus (LC) injections of synthetic tau fibrils is determined by the LC’s afferent and efferent connections. *Acta Neuropathol. (Berl.)* **130**, 349–362 (2015).

6. Narasimhan, S. *et al.* Pathological Tau Strains from Human Brains Recapitulate the Diversity of Tauopathies in Nontransgenic Mouse Brain. *J. Neurosci. Off. J. Soc. Neurosci.* **37**, 11406–11423 (2017).

7. Vergara, C. *et al.* Amyloid-β pathology enhances pathological fibrillary tau seeding induced by Alzheimer PHF in vivo. *Acta Neuropathol. (Berl.)* **137**, 397–412 (2019).

8. Holth, J. K. *et al.* The sleep-wake cycle regulates brain interstitial fluid tau in mice and CSF tau in humans. *Science* **363**, 880–884 (2019).

9. Miao, J. *et al.* Pathological Tau From Alzheimer’s Brain Induces Site-Specific Hyperphosphorylation and SDS- and Reducing Agent-Resistant Aggregation of Tau in vivo. *Front. Aging Neurosci.* **11**, 34 (2019).

10. Skachokova, Z. *et al.* Cerebrospinal fluid from Alzheimer’s disease patients promotes tau aggregation in transgenic mice. *Acta Neuropathol. Commun.* **7**, 72 (2019).

11. He, Z. *et al.* Transmission of tauopathy strains is independent of their isoform composition. *Nat. Commun.* **11**, 7 (2020).

12. Masuda-Suzukake, M. *et al.* Dextran sulphate-induced tau assemblies cause endogenous tau aggregation and propagation in wild-type mice. *Brain Commun.* **2**, fcaa091 (2020).
